# The Anesthesia Preparedeness Clinic (APC) triage score tool: an integrated electronic health record tool for improving resource allocation for preoperative care clinics

**DOI:** 10.1007/s10877-026-01438-8

**Published:** 2026-04-16

**Authors:** Minhthy N. Meineke, Alice Chien, Sophie W. Mawdsley, Megan Meyer, Brian H. Park, Elizabeth Kim, Rodney A. Gabriel

**Affiliations:** 1 Division of Perioperative Informatics, Department of Anesthesiology, University of California, San Diego, La Jolla, CA USA; 2 Department of Anesthesiology, University of California, San Diego, La Jolla, CA USA; 3 School of Medicine, University of California, San Diego, La Jolla, CA USA; 4 Depatment of Biomedical Informatics, University of California, San Diego Health, La Jolla, CA USA

**Keywords:** Electronic health record, Perioperative medicine, Anesthesiology

## Abstract

Pre-anesthesia clinics have been associated with improved patient outcomes, but with the rising surgical volume, there is a need to improve allocation of preoperative evaluations. We developed the Anesthesia Preparedeness Clinic (APC) Triage Score tool that was integrated into the electronic health record system and calculated a score based on preoperative factors. It was used to allocate preoperative evaluations for patients to either in-person, telehealth, or nursing phone screens. A retrospective cohort analysis was performed compare how resources were allocated prior to and after implementation (Pre-APC and Post-APC, respectively). There were a total of 9,986 and 10,487 surgical patients included in the analysis in the Pre-APC and Post-APC cohorts, respectively. The correlation coefficient between the triage score and American Society of Anesthesiologists Physical Status (ASA PS) score based on Spearmen test was 0.59 (*P* < 0.001). The median (quartiles) APC Triage Score in patients who were ASA PS 1 was 0 (0, 1.5), ASA PS 2 was 3.0 (1.0, 6.0), ASA PS 3 was 9.5 (4.5, 17.0) and ASA PS 4 was 21.0 (11.5, 29.5). Patients that received an in-person visit tended to have higher APC Triage Scores in the Post-APC versus Pre-APC cohort with a median (quartiles) score of 17.0 (12.0, 25.5) versus 10.0 (6.0, 19.5), respectively (*P* < 0.001). Patients that received a telehealth visit tended to have higher APC Triage Scores in the Post-APC versus Pre-APC cohort with a median (quartiles) score of 8.0 (5.0, 13.5) versus 7.0 (3.5, 13.0), respectively (*P* < 0.001). Finally, patients that received a phone visit tended to have a lower APC Triage Score in the Post-APC versus Pre-APC cohorts with a median (quartiles) score of 3.0 (1.0, 7.0) versus 4.0 (1.0, 8.5), respectively (*P* < 0.001). Our study demonstrated that implementation of the tool was associated with improved preoperative allocations.

**Supplementary Information:**

The online version contains supplementary material available at 10.1007/s10877-026-01438-8.

## Introduction

Pre-anesthesia clinics are outpatient services designed to assess patients’ medical history and anesthesia-related risks to optimize safety and minimize perioperative complications [1]. Depending on case complexity and institutional resources, clinic staffing can consist of anesthesiologists, advanced practice providers, and registered nurses (RN). These structured assessments involve medication reconciliation, review of medical records, physical examinations, diagnostic testing, and patient education - all of which inform anesthetic management. Pre-anesthesia clinics have been associated with improved patient outcomes, such as reduced inpatient length of stay, lower mortality, and enhanced planning for complex cases [1, 2]. Beyond clinical benefits, they also increase operational efficiency by reducing same-day surgical cancellations and in-person clinic consultation time [3, 4].

As surgical volume increases, hospitals face the challenge of safely and efficiently managing growing patient populations. Traditional in-person pre-operative evaluations, while thorough, are resource- and time-intensive. Improvements in perioperative safety and decreases in perioperative mortality suggest that low-risk patients may not require intensive evaluation [5, 6]. In response, hospitals have explored alternative triage strategies such as the use of structured patient questionnaires, phone-based screenings, and nurse consultations [7]. Implemented pre-operative assessment tools have demonstrated significant reductions in in-person consultations, though the majority rely on self-reported data, which could introduce variability [8, 9]. Meanwhile, machine learning-based triage models have shown promise triaging patients in retrospective studies, but have yet to demonstrate real-time improvements in clinic workflow [10, 11].

To address these limitations, UC San Diego’s pre-anesthesia clinic, the Anesthesia Preparedness Clinic (APC) developed and implemented the APC Triage Score tool, a risk stratification algorithm integrated into our electronic medical record system (Epic Systems, Verona, WI). This tool assigns every surgical patient a live electronic triage score based on certain International Classification of Disease-9 and − 10 diagnoses and medications, which are weighted according to risk, as established by consensus among attending anesthesiologists. The cumulative score reflects overall clinical complexity of the patient and informs triage decisions. Based on the patient’s APC triage score, they are either assigned an in-person (with an advanced practice provider and/or anesthesiologist), telehealth (with an advanced practice provider), RN phone evaluation or bypass the clinic if there is no RN access. This data-driven tool aims to streamline preoperative workflows, reduce unnecessary clinic visits, and improve resource allocation to the patients who need it most.

In this study, we aim to evaluate the performance and clinical utility of the APC Triage tool within a high-volume tertiary care center. Specifically, we conducted a pre-post implementation analysis to evaluate the associated changes in workflow. Our primary hypothesis was that following implementation, there would be a greater alignment between patient complexity and evaluation modality. We predicted that sicker patients would be more likely to have in-person/telemedicine visits, while healthier patients were more likely to be managed with RN phone calls.

## Methods

### Study population

The resulting dataset remained de-identified and did not contain sensitive patient-health information as defined by the institutional Human Research Protections Program, and, therefore, was exempt from the informed consent requirement and approved by our Institutional Review Board. Data were collected retrospectively from the electronic health record of our institution. In this retrospective cohort study, we compared two surgical cohorts during the Pre-APC period (prior to implementation of our APC Triage Scoring System) and the Post-APC period (after implementation of our APC Triage Scoring System). The APC Triage Score system was initiated on October 2024. We extracted surgical patients at our institution to represent both cohorts. The Pre-APC period included all surgical cases from August 1 to September 30, 2024 and the Post-APC period included all surgical cases from April 1 to June, 30 2025. We chose a time period several months after implementation to provide adequate time for stabilizing the workflow. Furthermore, there were no staffing changes in full-time equivalents in the APC during the time period for both cohorts. For patients that had multiple surgeries, we only included their first encounter.

### Description of the Anesthesia Preparedness Clinic

The APC’s purpose is to evaluate patients prior to surgery and identify any potential issues that need to be optimized or addressed to minimize perioperative morbidity/mortality and to improve the patient’s readiness to undergo anesthesia. APC is staffed with RNs and advanced practice practitioners (physician assistant and nurse practitioner) who are supervised by an attending anesthesiologist. The RN visit is a phone interview with the patient to confirm current medications, perform a basic anesthesia specific review of systems and provide preoperative and medication instructions. The advanced practice practitioner visit can be performed either via telehealth or in-person. Due to the expected increased complexity of the visit, there is more in-depth information gathering and evaluation of the patient’s comorbid conditions, in addition to a medication reconciliation and providing preoperative instructions.

### Development of the APC triage score tool

A group of attending anesthesiologists that staff the APC at University of California, San Diego were surveyed what diagnoses codes (present in medical history and problem list in the electronic health record) and medications were considered pertinent in triaging patients to the three levels of APC appointments (in-person versus telehealth versus RN phone screen). Once the list was compiled, the same group of 8 anesthesiologists were asked to assign a numeric value (1–10) to each factor. The values were averaged for each factor. See **Supplemental Table 1** for the complete list of factors and their assigned score. To avoid duplication, rules were applied to certain factors to avoid a falsely high APC triage score. For instance, hypertension or “taking an anti-hypertensive medication” yielded the same value. The APC triage tool underwent production in our electronic health record system (Epic Systems, Verona, WI). At the initiation of the APC Triage Score in October 2024, for one month, we continued to have an APC Charge RN triage APC appointments and confirm the accuracy of each patient’s APC triage score. During this process, we created a scale of 0-5.5 to be assigned RN phone screen, 6-14.5 to be assigned telehealth, ≥ 15 to be assigned an in-person visit. Based on individual chart review of inaccurate triage scores, we adjusted the tool by adding (i.e.: “developmental delay”) and removing factors (“chronic pain”), condensing the medications from individual medications to medication classes, and lastly adjusting the values of particular factors to reflect a more accurate score. After the trial, APC launched a new standard work that removed an APC Charge RN from performing triage duties and triaging appointments was based entirely on the APC triage tool. Thus, our cohort during the post-APC study period was based on the APC Triage Score tool itself without external validation from staff.

### Study objective

The objectives of this retrospective observational study were to: (1) correlate the APC Triage Scores with clinician-assigned American Society of Anesthesiologists Physical Status (ASA PS) scores; and (2) determine if patients receiving in-person and televideo health visits had higher APC Triage Scores while those receiving phone screens had lower APC Triage Scores when comparing Post-APC to Pre-APC study cohorts. This would suggest that the APC Triage Scoring system helped improve allocation of higher risk patients to higher level of preanesthesia evaluations (in-person and tele-health) while lower risk patients received lower level of preanesthesia evaluations (RN phone interview).

### Data collection

For all patients, we collected age at time of surgery, body mass index, ASA PS, type of APC visit (in-person, tele-health, phone interview, versus none), and the calculated APC Triage Score. The score was calculated retroactively from retrieving the relevant data from the electronic health record system.

### Statistical analysis

All statistical analyses were performed using R (Version 4.2.2). Chi-square test and Wilcoxon rank sum tests were used to calculate statistical significance between cohorts for categorical and continuous variables, respectively. Specifically, the APC Triage Score was reported as median and 25% and 75% quartiles and differences between categories were determined by Wilcoxon rank sum test. A *P* < 0.05 was considered statistically significant. A previous study had demonstrated that the square root of total medications was an accurate predictor of preoperative anesthesia clinic appointment duration [12]. As appointment duration may be directly correlated with the type of anesthesia visit required (in-person suggests longer time needed for a patient versus phone call visit), we also aimed to quantify the incremental benefit of predicting visit type with our score tool versus number of medications (square rooted). For this, we modeled the associations of these features with the outcome using logistic regression and reported the c-statistics. Spearman correlation coefficient was calculated to assess the correlation between APC Triage Score and ASA PS score. To adjust for the number of medications (square rooted), we used partial Spearman correlation.

To quantify feature importance of each of the elements included in the APC Triage Score tool for model performance, we conduct a multivariable logistic regression model, in which the dependent variable was allocation to an in-person visit and the independent variables were all elements of the APC Triage Score tool. Feature importance was then calculated for each variable based on its impact on the model’s area under the receiver operating characteristics curve.

Due to the cohorts occurring at the different time periods, we performed a segmented regression analysis of an interrupted time series to model trends in median APC Triage Score among those receiving an in-person pre-anesthesia visit during the: (1) pre-APC study period; (2) immediately when the post-APC period started; and (3) Post-APC study period. To perform a segmented regression, we utilized the following regression equation:   

$${\mathrm{Y}} = {\mathrm{b}}0\, + \,{\mathrm{b1T}} + {\mathrm{b2D}} + {\mathrm{b3X}}\, + \,{\mathrm{e}}.$$

where: (Y) = the outcome variable (the median APC Triage Score in one month for patients receiving an in-person visit); (T) = continuous variable which indicates time passed from the start of the observation period; (D) = a variable indicating observation collected before or after initiation of APC Triage Score; and (X) = a continuous variable indicating time passed since the start of the Post-APC study period. Statistically significant estimates for T would indicate a trend change in the outcome during the pre-APC period; for D would indicate an immediate change in the outcome when Post-APC period started; and for X would indicate a trend change in the outcome during the time period from start of the Post-APC period to end of study period.

## Results

### Study population

The APC Triage Score system was implemented in October of 2024. We extracted all surgical patient data from a time period (August to September 2024) prior to implementation (Pre-APC) and compared it to patient data from a time period (April to June 2025) after it was implemented (Post-APC). APC staffing remained consistent during both study periods. There were a total of 9,986 and 10,487 surgical patients included in the analysis in the Pre-APC and Post-APC cohorts, respectively (Table 1). The median (quartiles) calculated APC Triage Score for the Pre-APC and Post-APC cohorts were 5.5 (1.5, 13.0) versus 6.5 (2.0, 14.5), respectively (*P* < 0.001). A higher percentage of patients were seen as an in-person visit (3.2% versus 1.8%) and via tele-health visit (8.1% versus 5.8%) while less patients were allocated to a phone visit (19.3% versus 23.1%) in the Post-APC versus Pre-APC cohorts, respectively (*P* < 0.001). The percentage of surgical patients that did not receive an APC visit were similar in both cohorts.

**Table 1:** Distribution of characteristics between both cohorts: prior to operationalizing APC Triage Scoring system versus the time period when it was operationalized

	Prior to APC triage score implementation	Period when APC triage score was implemented	*P*-value
Total	9986	10,487	
Age (years), median [quartiles]	60 [46, 71]	60 [44, 70]	0.02
Sex, n (%)			0.08
Female	5261 (52.7)	5602 (53.4)	
Male	4717 (47.2)	4866 (46.4)	
Unknown	8 (0)	19 (0.1)	
BMI (kg/m2), median [quartiles]	26.6 [23.3, 30.6]	26.5 [23.1, 30.6]	0.33
ASA PS score, n (%)			< 0.001
1	506 (5.1)	529 (5.0)	
2	2671 (26.7)	2549 (24.3)	
3	2902 (29.1)	3404 (32.5)	
4	302 (3.0)	342 (3.3)	
5	18 (0.2)	13 (0.1)	
6	2 (0)	6 (0)	
unknown	3585 (35.9)	3644 (34.7)	
Calculated APC Triage Score, median [quartiles]	5.5 [1.5, 13.0]	6.5 [2.0, 14.5]	< 0.001
Type of APC visit, n (%)			< 0.001
In-Person	181 (1.8)	331 (3.2)	
Televideo	579 (5.8)	846 (8.1)	
Phone call	2309 (23.1)	2027 (19.3)	
No preoperative visit	6917 (69.3)	7283 (69.4)	

Abbreviations: APC, anesthesia preparedness clinic; ASA PS, American Society of Anesthesiologists Physical Status; BMI, body mass index

### The APC triage score is correlated with ASA PS score

**Fig. 1:**
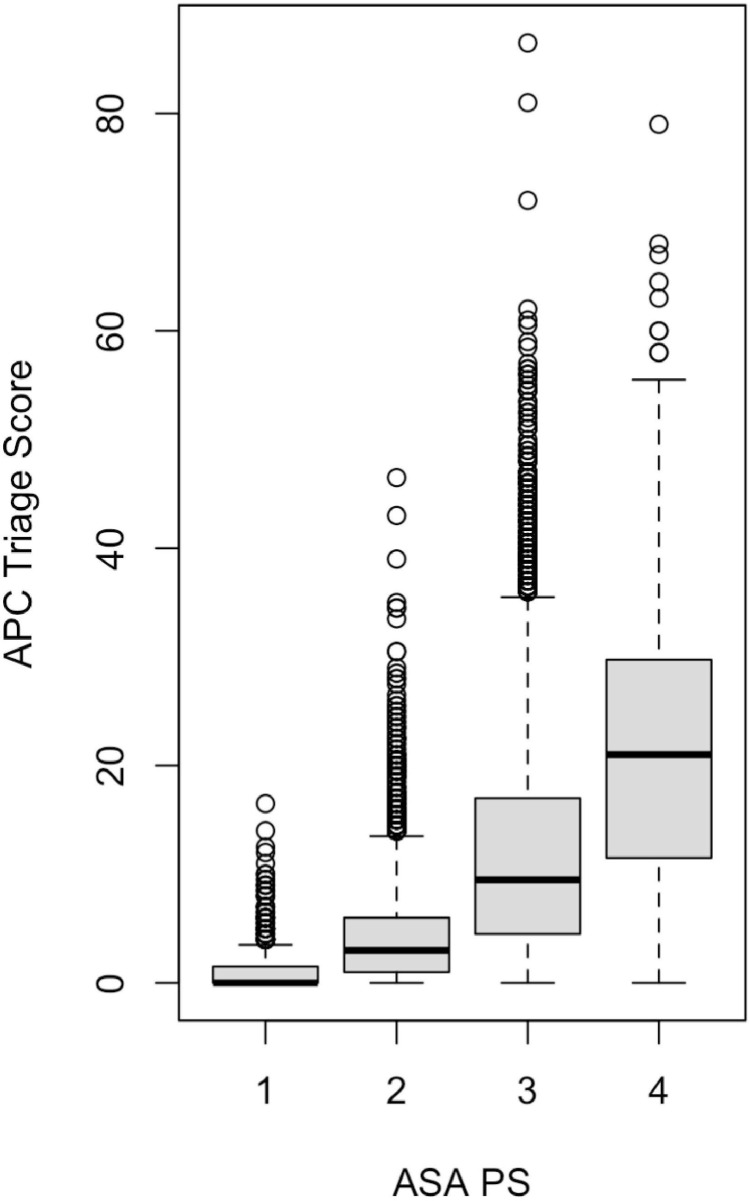
Box plot illustrating the distribution of the APC Triage Score for patients that are ASA PS 1–4. Abbreviations: APC, Anesthesia Preparedness Clinic; ASA PS, American Society of Anesthesiologists Physical Status

Next, we determined if the APC Triage Score correlated with assigned ASA PS scores. For this analysis, we removed all patients with unknown ASA PS scores. The correlation coefficient based on Spearmen test was 0.59 (*P* < 0.001). To assess the association between APC Triage Score and ASA PS scores while adjusting for the square root of the number of medications, we calculated the partial Spearman correlation, which was 0.49 (*P* < 0.001). The median (quartiles) APC Triage Score in patients who were ASA PS 1 was 0 (0, 1.5), ASA PS 2 was 3.0 (1.0, 6.0), ASA PS 3 was 9.5 (4.5, 17.0) and ASA PS 4 was 21.0 (11.5, 29.5) (Fig. 1).

### Implementation of the APC triage score was associated with improved allocation of preanesthesia clinic resources

**Fig. 2:**
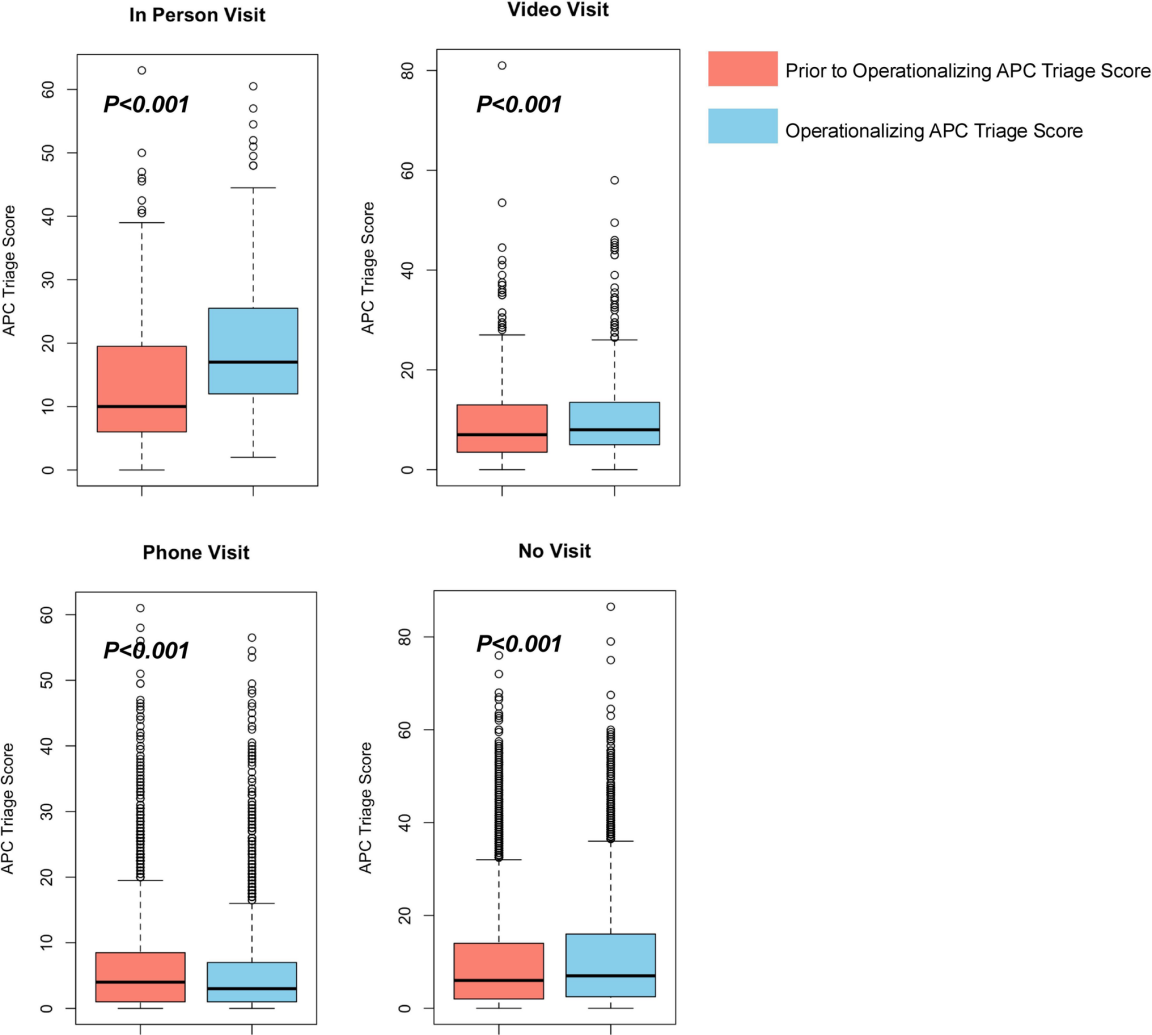
Box plots illustrating the distribution of the APC Triage score for patients in the Pre-APC and Post-APC cohorts in those who received a preoperative in-person, telehealth (video), phone visit (by registered nurse), or no visit. Abbreviations: APC, Anesthesia Preparedness Clinic

We then calculated the median APC Triage Scores among patients who received an in-person, tele-health, phone versus no visit between the pre-APC and post-APC cohorts (Fig. 2). Patients that received an in-person visit tended to have higher APC Triage Scores in the Post-APC versus Pre-APC cohort with a median (quartiles) score of 17.0 (12.0, 25.5) versus 10.0 (6.0, 19.5), respectively (*P* < 0.001). Patients that received a telehealth visit tended to have higher APC Triage Scores in the Post-APC versus Pre-APC cohort with a median (quartiles) score of 8.0 (5.0, 13.5) versus 7.0 (3.5, 13.0), respectively (*P* < 0.001). Finally, patients that received a phone visit tended to have a lower APC Triage Score in the Post-APC versus Pre-APC cohorts with a median (quartiles) score of 3.0 (1.0, 7.0) versus 4.0 (1.0, 8.5), respectively (*P* < 0.001). This suggests that implementation of the APC Triage Scoring System was associated with appropriate triaging of higher risk patients to in-person/tele-health screening and lower risk patients to phone screening.

To quantify the incremental improvement of the APC Triage Score compared to using number of medications, we calculate the c-statistic for measuring association of in-person visits, telemedicine visits, and phone visit with the: (1) APC Triage Score alone; (2) square root of number of medications alone; versus (3) combined APC Triage score alone and square root of the number of medications. For in-person visits, the c-statistics for these model configurations were 0.849, 0.701, and 0.844, respectively. For telehealth visits, the c-statistics were 0.752, 0.622, and 0.746, respectively. For phone visits, the c-statistics were 0.774, 0.656, and 0.771, respectively.

**Fig. 3:**
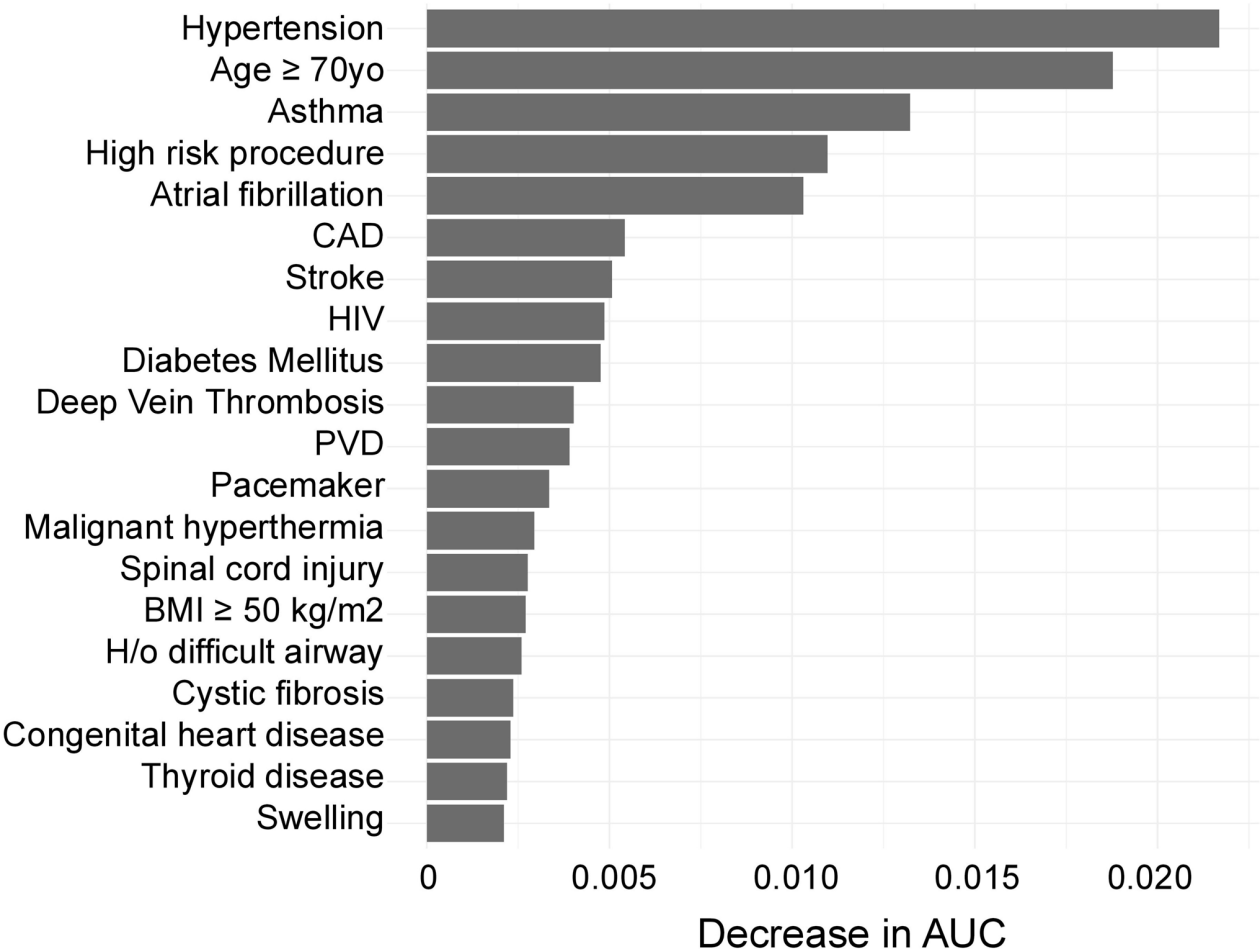
Feature importance plot illustrating the top 20 variables with the highest impact on model performance based on area under the receiver operating characteristics curve. Abbreviations: BMI, body mass index; CAD, coronary artery disease; HIV, human immunodeficiency virus; PVD, peripheral vascular disease

We next performed a multivariable logistic regression to calculate the association of each APC Triage Score variable for each patient with assignment of an in-person visit. Figure 3 illustrates the top 20 most important features impacting model performance, in which, hypertension, age, asthma, planned high risk procedure, and atrial fibrillation had the highest importance.

**Fig. 4:**
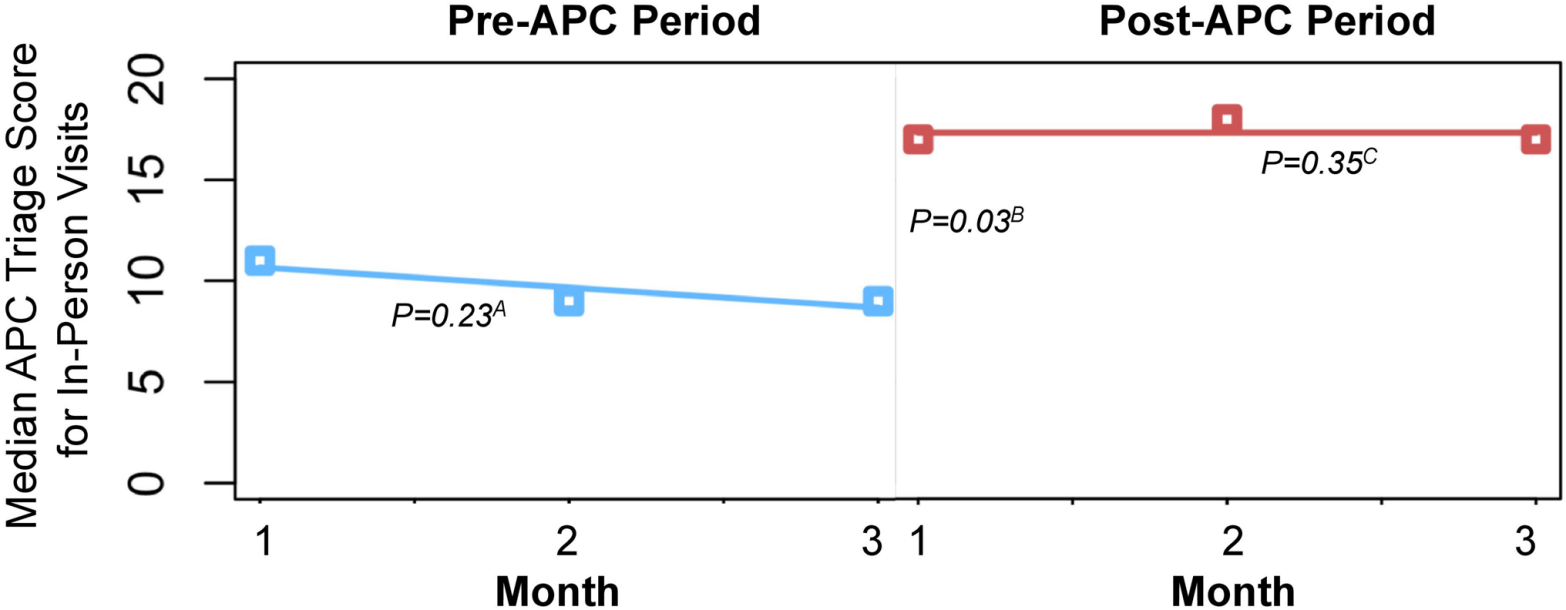
Results from segmented regression analysis using the data during the Pre-APC and Post-APC study population. Each study period contained 3 months of surgical patients. The median APC Triage Score per month are reported for each cohort. **A**) There was no statistically significant trend in changes of in the median APC Triage Score during pre-APC interval (*p* = 0.23); **B**) there was a statistically significant increase in the median APC Triage Score immediately at the beginning of the post-APC Triage score period (*p* = 0.03); and **C**) there was no statistically significant trend in the median APC Triage Score during the post-APC study period (*p* = 0.35). Abbreviations: APC, Anesthesia Preparedness Clinic

We subsequently performed a segmented regression analysis using the data during the Pre-APC and Post-APC study population (Fig. 4). Each study period contained 3 months of surgical patients. The median APC Triage Score per month are reported for each cohort. There was no statistically significant trend in changes of in the median APC Triage Score during pre-APC interval (*p* = 0.23). Furthermore, there was no statistically significant trend in the median APC Triage Score during the post-APC study period (*p* = 0.35). However, there was a statistically significant increase in the median APC Triage Score immediately at the beginning of the post-APC Triage score period (*p* = 0.03).

### Same-day cancellations

There were 249 (2.5%) same day cancellations (defined as cases cancelled preoperatively on day of surgery due to inadequate medical clearance or preoperative work) during the pre-APC study period. There were 199 (1.9%) same day cancellations during the post-APC study period. The difference was statistically significant (*P* = 0.003).

## Discussion

In this retrospective analysis of 20,473 adult surgical patients, we demonstrated that the implementation of the APC Triage Score system was associated with improved triaging of higher risk patients to in-person/tele-health screening and lower risk patients to phone interview. This was demonstrated based on the higher median APC Triage Score values in the in-person and tele-health assessments and lower median APC Triage Score values in the phone interviews during the Post-APC cohort. Furthermore, the APC Triage Score was correlated with clinician-assigned ASA PS scores. We also demonstrated that the tool had stronger associations with triaging compared to total medications prescribed to patients, which has previously been shown to be an accurate predictor for visit time duration at a pre-anesthesia clinic [12]. These findings suggest that this automated APC Triage Score tool may be useful for optimizing the allocation of different levels of preanesthesia clinic assessments in the setting of limiting staffing resources and rising surgical volumes. We demonstrated that with higher scoring, clinically complex patients were preferentially routed to in-person and tele-health visits with anesthesiologists or advanced practice providers, whereas low-scoring patients were managed through nurse phone interviews. While we demonstrated that same day cancellations were reduced in the post-APC period, future prospective studies are needed to determine if such allocations produce a measurable reduction in same-day cancellations and clinic congestion.

The study shows that an automated, data-driven algorithm can improve perioperative resource allocation with minimal manual effort. By using diagnosis codes and high-risk medication lists already available in the electronic health record, the score functions as a scalable surrogate to approximate patient complexity without additional patient surveys. This allows lower-risk patients to safely bypass in-person clinic visits while focusing time and attention on high-risk cases, improving both operational efficiency and patient safety. Because the APC Triage Score system is integrated directly into the electronic health record system, it can be deployed across all surgical specialties without adding survey burden or duplicative documentation, thereby reducing clinician workload. Given that manually reviewing and triaging patients is resource-intensive, the APC Triage Score system is a promising way to streamline preoperative screening while maintaining high-quality perioperative care without using sophisticated computational needs associated with more advanced machine learning algorithms [13, 14].

Earlier triage programs such as Pre-PAS (Pre-operative Patient-centred Anaesthesia System) and PACMAN (Pre-operative triage procedure to streaMline elective patieNts) rely on nurse- or patient-completed questionnaires to steer complex patients toward anesthesiologist review, successfully reducing face-to-face consultations but at the cost of additional administrative steps and depended on self-reported patient information [8, 9]. More recent approaches bypass surveys and instead leverage structured chart data. For example, PCATS (Patient-Centered Anesthesia Triage System) uses factors like prescription counts, body mass index, age, and surgical complexity to recommend tele-health versus clinic visits, while the Pre-Anesthesia AI Tool applies Bayesian deep learning across thirty electronic health record variables to infer triage decisions [11]. Our study builds on these tools by validating a fully electronic health record integrated, real-time algorithm that depends on specific clinician-derived diagnosis codes and a short list of flagged medications to stratify patients. The automation and standardization, along with electronic health record integration, allows for tangible operational benefits in workflow.

The key strengths of this study include system-wide deployment across all elective surgical specialties and seamless integration within the electronic health record platform, which allowed for automatic triaging without additional manual data entry. This widescale use highlights the practicality and scalability in a real clinical setting. However, given that this was a single-center study, there are limitations to its external validity and effectiveness at other institutions that may have alternative electronic health record platforms or workflows. Additionally, clinicians were aware of patients’ APC Triage Scores while scheduling, potentially introducing allocation bias. Our analyses also focused on workflow measures as endpoints rather than patient satisfaction or perioperative complications. Future multicenter studies will be helpful in confirming these findings and determining whether the APC Triage Score tool translates into improved patient experiences and outcomes.

Although the APC Triage Score system was developed and validated at our institution, it is likely to translate well to hospitals that use integrated electronic health records and maintain consistent diagnostic coding practices. Adaptations will be required for centers that treat specialized populations (e.g. neonatal or highly complex pediatric populations), and the medication weight list must be adjusted to reflect local formularies. Because coding practices and electronic health records can vary widely across institutions, the scoring system may need recalibration to ensure consistent data capturing and scoring.

The APC Triage Score safely and effectively aligned clinician expertise with patient needs, streamlined preoperative workflows, and was associated with decreased same-day cancellations. Higher-risk patients were more likely to receive in-person or telehealth visits, while lower-risk patients were evaluated with a phone interview. With broader adoption and rigorous external validation, the APC Triage Score tool has the potential to standardize triage across diverse surgical environments, as well as sustain high-quality, efficient perioperative care. These findings demonstrate that a data-driven approach to pre-operative evaluation can meaningfully optimize resources while maintaining patient safety, providing a model for broader adoptions in other surgical centers.

## Supplementary Information

Below is the link to the electronic supplementary material.

Supplementary Material 1

## Data Availability

De-identified data can be made available with appropriate data use agreements with our institution.
