# Supplementary material for: The Anesthesia Preparedeness Clinic (APC) triage score tool: an integrated electronic health record tool for improving resource allocation for preoperative care clinics
Source: J Clin Monit Comput. 2026 Apr 16;40(4):923–32. doi: 10.1007/s10877-026-01438-8 (PMC13391741; doi:10.1007/s10877-026-01438-8)
Supplement: Supplementary file 1 — Supplementary Material 1 [file 10877_2026_1438_MOESM1_ESM.docx]

| **Supplemental Table 1.** Conditions and corresponding electronic health record code or data source for APC Triage Score tool. | | |
| --- | --- | --- |
| **Condition** | **Codes Used** | **APC Score** |
| One or more of the patient's procedures is categorized as high risk | institution-specific grouper for this classification | 5 |
| Patient has a BMI between 40-50 | structured data from electronic health record | 3 |
| Patient has a BMI greater than 50 | structured data from electronic health record | 5 |
| Patient is pregnant | structured data from electronic health record | 1 |
| Patient was hospitalized within the last three months | patient response to surgery scheduler survey | 3 |
| Patient has a difficult airway or other anesthesia problem | patient response to surgery scheduler survey | 2 |
| Patient has a low activity tolerance | patient response to surgery scheduler survey | 3 |
| Patient age is >= 70 | structured data from electronic health record | 2 |
| Patient has a history of pulmonary hypertension | I27.0, I27.2, I27.20, I27.21, I27.22, I27.23, I27.24, I27.29, P29.3, P29.30 | 5 |
| Patient has a history of lung cancer | C34, C34.1, C34.2, C34.3, C34.8, C34.9, C34.00, C34.01, C34.02, C34.11, C34.12, C34.0, C34.30, C34.31, C34.32, C34.80, C34.81, C34.82, C34.90, C34.92, C34.91, C34.10 | 2.5 |
| Patient has a history of renal insufficiency | N17, N17.0, N17.1, N17.2, N17.8, N17.9 | 2.5 |
| Patient has a history of asthma or is taking an antiasthmatic medication | J45, J45.2, J45.20, J45.21, J45.22, J45.3, J45.30, J45.31, J45.32, J45.4, J45.40, J45.41, J45.42, J45.5, J45.50, J45.51, J45.52, J45.9, J45.90, J45.99, J45.901, J45.902, J45.909 | 1 |
| Patient has a history of COPD | J44, J44.0, J44.1, J44.81, J44.89, J44.9 | 3 |
| Patient has a history of anemia | D50, D50.0, D50.1, D50.8, D50.9, D53, D53.0, D53.1, D53.2, D53.8, D53.9 | 3 |
| Patient has a history of coagulopathy | D65,D66, D67, D68, D68.0, D68.00, D68.01, D68.020, D68.021, D68.022, D68.023, D68.029 D68.03, D68.04, D68.09, D69.1, D68.2, D68.3, D68.31, D68.311, D68.312, D68.318, D68.32, D68.4, D68.5, D68.51, D68.52, D68.59, D68.6, D68.61, D68.62, D68.69, D68.8, D68.9, D69, D69.0, D69.2, D69.3, D69.4, D69.41, D69.42, D69.49, D69.5, D69.51, D69.59, D69.6, D69.8, D69.9 | 3 |
| Patient has a history of DVT | I82, I82.0, I82.1, I82.2, I82.21, I82.22, I82.210, I82.211, I82.220, I82.221, I82.290, I82.291, I82.3, I82.4, I82.40, I82.401, I82.402, I82.403, I82.409, I82.41, I82.411, I82.412, I82.413, I82.419, I82.42, I82.421, I82.423, I82.429, I82.43, I82.431, I82.432, I82.433, I82.439, I82.44, I82.441, I82.442, I82.443, I82.449, I82.5,I82.50, I82.501, I82.502, I82.503, I82.509, I82.51, I82.511, I82.512, I82.513, I82.519, I82.52, I82.521, I82.522, I82.523, I82.529, I82.53, I82.531, I82.532, I82.533, I82.539, I82.54, I82.541, I82.542, I82.543, I82.549, I82.551, I82.552, I82.553, I82.559, I82.561, I82.562, I82.563, I82.569, I82.59, I82.591, I82.592, I82.593, I82.599, I82.6, I82.60, I82.601, I82.602, I82.603, I82.609, I82.61, I82.611, I82.612, I82.613, I82.619, I82.62, I82.621, I82.622, I82.623, I82.629, I82.70, I82.701, I82.702, I82.703, I82.709, I82.71, I82.711, I82.712, I82.713, I82.719, I82.72, I82.721, I82.722, I82.723, I82.729, I82.8, I82.81, I82.811, I82.812, I82.813, I82.819, I82.89, I82.891, I82.890, I82.9, I82.90, I82.91 | 3 |
| Patient has a history of hepatitis | B16, B16.1, B16.2, B16.9, B17, B17.0, B17.1, B17.11, B17.2, B17.8, B17.9, B18, B18.0, B18.1, B18.2, B18.8, B18.9, B19, B19.0, B19.10, B19.11, B19.2, B19.21, B19.9 | 3 |
| Patient has a history of esophageal varices | I85, I85.0, I85.00, I85.01, I85.11, I85.10 | 3 |
| Patient has a history of seizures or is taking an anticonvulsant medication | G40, G40.0, G40.00, G40.001, G40.009, G40.01, G40.011, G40.019, G40.1, G40.10, G40.101, G40.109, G40.11, G40.111, G40.119, G40.2, G40.20, G40.201, G40.209, G40.21, G40.211, G40.219, G40.3, G40.30, G40.301, G40.309, G40.31, G40.311, G40.319, G40.4, G40.40, G40.401, G40.409, G40.41, G40.411, G40.419, G40.42, G40.5, G40.50, G40.501, G40.509, G40.8, G40.80, G40.801, G40.802, G40.803, G40.804, G40.81, G40.811, G40.812, G40.813, G40.814, G40.82, G40.821, G40.822, G40.823, G40.824, G40.833, G40.834, G40.89, G40.9, G40.90, G40.901, G40.909, G40.91, G40.911, G40.919, G40.A, G40.A0, G40.A01, G40.A09, G40.A1, G40.A11, G40.A19, G40.B, G40.B0, G40.B01, G40.B09, G40.B1, G40.B11, G40.B19, G40.C01, G40.C09, G40.C11, G40.C19 | 1 |
| Patient has a history of valvular disorders | I34, I34.0, I34.1, I34.2, I34.8, I34.81, I34.89, I34.9, I35, I35.0, I35.1, I35.2, I35.8, I35.9, I36, I36.0, I36.1, I36.2, I36.8, I36.9, I37, I37.0, I37.1, I37.2, I37.8, I37.9 | 4 |
| Patient has a history of pulmonary embolism | I26, I26.0, I26.01, I26.02, I26.09, I26.9, I26.92, I26.90, I26.93, I26.94, I26.99 | 4 |
| Patient has a history of sickle-cell anemia | D57, D57.0, D57.00, D57.01, D57.02, D57.03, D57.04, D57.09, D57.1, D57.2, D57.20, D57.21, D57.211, D57.212, D57.213, D57.214, D57.218, D57.219, D57.3, D57.4, D57.40, D57.41, D57.411, D57.412, D57.413, D57.414, D57.418, D57.419, D57.42, D57.431, D57.432, D57.433, D57.434, D57.438, D57.439, D57.44, D57.451, D57.452, D57.453, D57.454, D57.458, D57.459, D57.8, D57.80, D57.81, D57.811, D57.812, D57.813, D57.814, D57.818, D57.819 | 6 |
| Patient has a history of brain tumor | C71, C71.0, C71.1, C71.2, C71.3, C71.4, C71.5, C71.6, C71.7, C71.8, C71.9 | 5 |
| Patient has a history of heart failure | I50, I50.1, I50.2, I50.20, I50.21, I50.22, I50.23, I50.3, I50.30, I50.31, I50.32, I50.33, I50.4, I50.41, I50.42, I50.43, I50.8, I50.81, I50.82,  I50.83, I50.84, I50.89, I50.810, I50.811, I50.812, I50.813, I50.814, I50.9 | 5 |
| Patient has a history of liver cirrhosis | K70.0, K70.1, K70.10, K70.11, K70.2, K70.3, K70.30, K70.31, K74.6, K74.60, K74.69, K70.4, K70.40, K70.41, K70.9, K70 | 5 |
| Patient has a history of substance abuse | F10, F10.1, F10.10, F10.11, F10.12, F10.120, F10.121, F10.129, F10.130, F10.131, F10.132, F10.139, F10.14, F10.15, F10.150, F10.151, F10.159, F10.18, F10.180, F10.181, F10.182, F10.188, F10.19, F10.2, F10.20, F10.21, F10.22, F10.220, F10.221, F10.229, F10.23, F10.230, F10.231, F10.232, F10.239, F10.24, F10.25, F10.250, F10.251, F10.259, F10.26, F10.27, F10.28, F10.280, F10.281, F10.282, F10.288, F10.29, F10.9, F10.90, F10.91, F10.92, F10.920, F10.921, F10.929, F10.930, F10.931, F10.932, F10.939, F10.94, F10.95, F10.950, F10.951, F10.959, F10.96, F10.97, F10.98, F10.980, F10.981, F10.982, F10.988, F10.99, F11, F11.1, F11.10, F11.11, F11.12, F11.120, F11.121, F11.122, F11.129, F11.13, F11.14, F11.15, F11.150, F11.151, F11.159, F11.18, F11.181, F11.182, F11.188, F11.19, F11.2, F11.20, F11.21, F11.22, F11.220, F11.221, F11.222, F11.229, F11.23, F11.24, F11.25, F11.250, F11.251, F11.259, F11.28, F11.281, F11.282, F11.288, F11.29, F11.9, F11.90, F11.91, F11.92, F11.920, F11.921, F11.922, F11.929, F11.93, F11.94, F11.95, F11.950, F11.951, F11.959, F11.98, F11.981, F11.982, F11.988, F11.99, F12, F12.1, F12.10, F12.11, F12.12, F12.120, F12.121, F12.122, F12.129, F12.13, F12.15, F12.150, F12.151, F12.159, F12.18, F12.180, F12.188, F12.19, F12.2, F12.20, F12.21, F12.22, F12.220, F12.221, F12.222, F12.229, F12.23, F12.25, F12.250, F12.251, F12.259, F12.28, F12.280, F12.288, F12.29, F12.9, F12.90, F12.91, F12.92, F12.920, F12.921, F12.922, F12.929, F12.93, F12.95, F12.950, F12.951, F12.959, F12.98, F12.980, F12.988, F12.99, F13, F13.1, F13.10, F13.11, F13.12, F13.120, F13.121, F13.129, F13.130, F13.131, F13.132, F13.139, F13.14, F13.15, F13.150, F13.151, F13.159, F13.18, F13.180, F13.181, F13.182, F13.188, F13.19, F13.2, F13.20, F13.21, F13.22, F13.220, F13.221, F13.229, F13.23, F13.230, F13.231, F13.232, F13.239, F13.24, F13.25, F13.250, F13.251, F13.259, F13.26, F13.27, F13.28, F13.280, F13.281, F13.282, F13.288, F13.29, F13.9, F13.90, F13.91, F13.92, F13.920, F13.921, F13.929, F13.93, F13.930, F13.931, F13.932, F13.939, F13.94, F13.95, F13.950, F13.951, F13.959, F13.96, F13.97, F13.98, F13.980, F13.981, F13.982, F13.988, F13.99, F14, F14.1, F14.10, F14.11, F14.12, F14.120, F14.121, F14.122, F14.129, F14.13, F14.14, F14.15, F14.150, F14.151, F14.159, F14.18, F14.180, F14.181, F14.182, F14.188, F14.19, F14.2, F14.20, F14.21, F14.22, F14.220, F14.221, F14.222, F14.229, F14.23, F14.24, F14.25, F14.250, F14.251, F14.259, F14.28, F14.280, F14.281, F14.282, F14.288, F14.29, F14.9, F14.90, F14.91, F14.92, F14.920, F14.921, F14.922, F14.929, F14.93, F14.94, F14.95, F14.950, F14.951, F14.959, F14.98, F14.980, F14.981, F14.982, F14.988, F14.99, F15, F15.1, F15.10, F15.11, F15.12, F15.120, F15.121, F15.122, F15.129, F15.13, F15.14, F15.15, F15.150, F15.151, F15.159, F15.18, F15.180, F15.181, F15.182, F15.188, F15.19, F15.2, F15.20, F15.21, F15.22, F15.220, F15.221, F15.222, F15.229, F15.23, F15.24, F15.25, F15.250, F15.251, F15.259, F15.28, F15.280, F15.281, F15.282, F15.288, F15.29, F15.9, F15.90, F15.91, F15.92, F15.920, F15.921, F15.922, F15.929, F15.93, F15.94, F15.95, F15.950, F15.951, F15.959, F15.98, F15.980, F15.981, F15.982, F15.988, F15.99, F16, F16.1, F16.10, F16.11, F16.12, F16.120, F16.121, F16.122, F16.129, F16.14, F16.15, F16.150, F16.151, F16.159, F16.18, F16.180, F16.183, F16.188, F16.19, F16.2, F16.20, F16.21, F16.22, F16.220, F16.221, F16.229, F16.24, F16.25, F16.250, F16.251, F16.259, F16.28, F16.280, F16.283, F16.288, F16.29, F16.9, F16.90, F16.91, F16.92, F16.920, F16.921, F16.929, F16.94, F16.95, F16.950, F16.951, F16.959, F16.98, F16.980, F16.983, F16.988, F16.99, F17, F17.2, F17.20, F17.200, F17.201, F17.203, F17.208, F17.209, F17.21, F17.210, F17.211, F17.213, F17.218, F17.219, F17.22, F17.220, F17.221, F17.223, F17.228, F17.229, F17.29, F17.290, F17.291, F17.293, F17.298, F17.299, F18, F18.1, F18.10, F18.11, F18.12, F18.120, F18.121, F18.129, F18.14, F18.15, F18.150, F18.151, F18.159, F18.17, F18.18, F18.180, F18.188, F18.19, F18.2, F18.20, F18.21, F18.22, F18.220, F18.221, F18.229, F18.24, F18.25, F18.250, F18.251, F18.259, F18.27, F18.28, F18.280, F18.288, F18.29, F18.9, F18.90, F18.91, F18.92, F18.920, F18.921, F18.929, F18.94, F18.95, F18.950, F18.951, F18.959, F18.97, F18.98, F18.980, F18.988, F18.99, F19, F19.1, F19.10, F19.11, F19.12, F19.120, F19.121, F19.122, F19.129, F19.130, F19.131, F19.132, F19.139, F19.14, F19.15, F19.150, F19.151, F19.159, F19.16, F19.17, F19.18, F19.180, F19.181, F19.182, F19.188, F19.19, F19.2, F19.20, F19.21, F19.22, F19.220, F19.221, F19.222, F19.229, F19.23, F19.230, F19.231, F19.232, F19.239, F19.24, F19.25, F19.250, F19.251, F19.259, F19.26, F19.27, F19.28, F19.280, F19.281, F19.282, F19.288, F19.29, F19.9, F19.90, F19.91, F19.92, F19.920, F19.921, F19.922, F19.929, F19.93, F19.930, F19.931, F19.932, F19.939, F19.94, F19.95, F19.950, F19.951, F19.959, F19.96, F19.97, F19.98, F19.980, F19.981, F19.982, F19.988, F19.99, F24 | 0.5 |
| Patient has a history of sepsis | A40, A40.1, A40.3, A40.8, A40.9, A41, A41.0, A41.01, A41.02, A41.1, A41.2, A41.3, A41.4, A41.5, A41.50, A41.51, A41.52, A41.53, A41.54, A41.59, A41.8, A41.81, A41.89, A41.9 | 3 |
| Patient has a history of Peripheral Vascular Disease | I70, I70.0, I70.1, I70.2, I70.20, I70.201, I70.202, I70.203, I70.208, I70.209, I70.21, I70.211, I70.212, I70.213, I70.218, I70.219, I70.22, I70.221, I70.222, I70.223, I70.228, I70.229, I70.23, I70.231, I70.232, I70.233, I70.234, I70.235, I70.238, I70.239, I70.24, I70.241, I70.242, I70.243, I70.244, I70.245, I70.248, I70.249, I70.25, I70.26, I70.261, I70.262, I70.263, I70.268, I70.269, I70.29, I70.291, I70.292, I70.293, I70.298, I70.299, I70.3, I70.30, I70.301, I70.302, I70.303, I70.308, I70.309, I70.31, I70.311, I70.312, I70.313, I70.318, I70.319, I70.32, I70.321, I70.322, I70.323, I70.328, I70.329, I70.33, I70.331, I70.332, I70.333, I70.334, I70.335, I70.338, I70.339, I70.34, I70.341, I70.342, I70.343, I70.344, I70.345, I70.348, I70.349, I70.35, I70.36, I70.361, I70.362, I70.363, I70.368, I70.369, I70.39, I70.391, I70.392, I70.393, I70.398, I70.399, I70.4, I70.40, I70.401, I70.402, I70.403, I70.408, I70.409, I70.41, I70.411, I70.412, I70.413, I70.418, I70.419, I70.42, I70.421, I70.422, I70.423, I70.428, I70.429, I70.43, I70.431, I70.432, I70.433, I70.434, I70.435, I70.438, I70.439, I70.44, I70.441, I70.442, I70.443, I70.444, I70.445, I70.448, I70.449, I70.45, I70.46, I70.461, I70.462, I70.463, I70.468, I70.469, I70.49, I70.491, I70.492, I70.493, I70.498, I70.499, I70.5, I70.50, I70.501, I70.502, I70.503, I70.508, I70.509, I70.51, I70.511, I70.512, I70.513, I70.518, I70.519, I70.52, I70.521, I70.522, I70.523, I70.528, I70.529, I70.53, I70.531, I70.532, I70.533, I70.534, I70.535, I70.538, I70.539, I70.54, I70.541, I70.542, I70.543, I70.544, I70.545, I70.548, I70.549, I70.55, I70.56, I70.561, I70.562, I70.563, I70.568, I70.569, I70.59, I70.591, I70.592, I70.593, I70.598, I70.599, I70.6, I70.60, I70.601, I70.602, I70.603, I70.608, I70.609, I70.61, I70.611, I70.612, I70.613, I70.618, I70.619, I70.62, I70.621, I70.622, I70.623, I70.628, I70.629, I70.63, I70.631, I70.632, I70.633, I70.634, I70.635, I70.638, I70.639, I70.64, I70.641, I70.642, I70.643, I70.644, I70.645, I70.648, I70.649, I70.65, I70.66, I70.661, I70.662, I70.663, I70.668, I70.669, I70.69, I70.691, I70.692, I70.693, I70.698, I70.699, I70.7, I70.70, I70.701, I70.702, I70.703, I70.708, I70.709, I70.71, I70.711, I70.712, I70.713, I70.718, I70.719, I70.72, I70.721, I70.722, I70.723, I70.728, I70.729, I70.73, I70.731, I70.732, I70.733, I70.734, I70.735, I70.738, I70.739, I70.74, I70.741, I70.742, I70.743, I70.744, I70.745, I70.748, I70.749, I70.75, I70.76, I70.761, I70.762, I70.763, I70.768, I70.769, I70.79, I70.791, I70.792, I70.793, I70.798, I70.799, I70.8, I70.9, I70.90, I70.91, I70.92, I71, I71.0, I71.00, I71.01, I71.010, I71.011, I71.012, I71.019, I71.02, I71.03, I71.1, I71.10, I71.11, I71.12, I71.13, I71.2, I71.20, I71.21, I71.22, I71.23, I71.3, I71.30, I71.31, I71.32, I71.33, I71.4, I71.40, I71.41, I71.42, I71.43, I71.5, I71.50, I71.51, I71.52, I71.6, I71.60, I71.61, I71.62, I71.8, I71.9, I72, I72.0, I72.1, I72.2, I72.3, I72.4, I72.5, I72.6, I72.8, I72.9, I73, I73.0, I73.00, I73.01, I73.1, I73.8, I73.81, I73.89, I73.9, I74, I74.0, I74.01, I74.09, I74.1, I74.10, I74.11, I74.19, I74.2, I74.3, I74.4, I74.5, I74.8, I74.9, I75, I75.0, I75.01, I75.011, I75.012, I75.013, I75.019, I75.02, I75.021, I75.022, I75.023, I75.029, I75.8, I75.81, I75.89, I76, I77, I77.0, I77.1, I77.2, I77.3, I77.4, I77.5, I77.6, I77.7, I77.70, I77.71, I77.72, I77.73, I77.74, I77.75, I77.76, I77.77, I77.79, I77.8, I77.81, I77.810, I77.811, I77.812, I77.819, I77.82, I77.89, I77.9, I78, I78.0, I78.1, I78.8, I78.9, I79, I79.0, I79.1, I79.8, I80.3, I82.91, I87.8, I99.8, I99.9 | 3.5 |
| Patient has a history of Developmental Delay | F01, F01.5, F01.50, F01.51, F01.511, F01.518, F01.52, F01.53, F01.54, F02, F02.8, F02.80, F02.81, F02.811, F02.818, F03, F03.9, F03.90, F03.91, F03.911, F03.918, F03.92, F03.93, F03.94, Q90, Q90.0, Q90.1, Q90.2, Q90.9 | 5 |
| Patient has a history of hypertension or is taking an antihypertensive medication | I10, I15.8, I15.9, I16, I16.1, I16.0, I16.9 | 1 |
| Patient has a history of thyroid disorder | E00, E01, E02, E04, E05, E06, E07, E00.0, E00.1, E00.9, E01.0, E01.1, E01.2, E01.8, E04.0, E04.1, E04.2, E04.8, E04.9, E05.0, E05.00, E05.01, E05.10, E05.11, E05.20, E05.21, E05.30, E05.31, E05.40, E05.41, E05.80, E05.81, E05.90, E05.91 | 2 |
| Patient has a history of Diabetes Type 1 or Type 2, or is taking an antidiabetic medication | E10, E10.1, E10.10, E10.11, E10.2, E10.21, E10.22, E10.29, E10.3, E10.31, E10.311, E10.319, E10.32, E10.321, E10.3211, E10.3212, E10.3213, E10.3219, E10.329, E10.3291, E10.3292, E10.3293, E10.3299, E10.33, E10.331, E10.3311, E10.3312, E10.3313, E10.3319, E10.339, E10.3391, E10.3392, E10.3393, E10.3399, E10.34, E10.341, E10.3411, E10.3412, E10.3413, E10.3419, E10.349, E10.3491, E10.3492, E10.3493, E10.3499, E10.35, E10.351, E10.3511, E10.3512, E10.3513, E10.3519, E10.352, E10.3521, E10.3522, E10.3523, E10.3529, E10.353, E10.3531, E10.3532, E10.3533, E10.3539, E10.354, E10.3541, E10.3542, E10.3543, E10.3549, E10.355, E10.3551, E10.3552, E10.3553, E10.3559, E10.359, E10.3591, E10.3592, E10.3593, E10.3599, E10.36, E10.37, E10.39, E10.4, E10.40, E10.41, E10.42, E10.43, E10.44, E10.49, E10.5, E10.51, E10.52, E10.59, E10.6, E10.61, E10.610, E10.618, E10.62, E10.620, E10.621, E10.622, E10.628, E10.63, E10.630, E10.638, E10.64, E10.641, E10.649, E10.65, E10.69, E10.8, E10.9, E11, E11.0, E11.00, E11.01, E11.10, E11.11, E11.2, E11.22, E11.29, E11.21, E11.3, E11.31, E11.311, E11.319, E11.32, E11.321, E11.3211, E11.3212, E11.3213, E11.3219, E11.329, E11.3291, E11.3292, E11.3293, E11.3299, E11.33, E11.331, E11.3311, E11.3312, E11.3313, E11.3319, E11.339, E11.3391, E11.3392, E11.3393, E11.3399, E11.34, E11.341, E11.3411, E11.3412, E11.3413, E11.3419, E11.349, E11.3491, E11.3492, E11.3493, E11.3499, E11.35, E11.351, E11.3511, E11.3512, E11.3513, E11.3519, E11.359, E11.3591, E11.3592, E11.3593, E11.3599, E11.352, E11.3521, E11.3522, E11.3523, E11.3529, E11.353, E11.3531, E11.3532, E11.3533, E11.3539, E11.354, E11.3541, E11.3542, E11.3543, E11.3549, E11.355, E11.3551, E11.3552, E11.3553, E11.3559, E11.36, E11.37, E11.37X1, E11.37X2, E11.37X3, E11.37X9, E11.39, E11.4, E11.40, E11.41, E11.42, E11.43, E11.44, E11.49, E11.5, E11.51, E11.52, E11.59, E11.6, E11.61, E11.62, E11.63, E11.64, E11.65, E11.69, E11.8, E11.9, | 1.5 |
| Patient has a history of Liver Transplant | 14909, 123846, 165217, 226851, 228349, 281919, 345732, 392989, 393379, 417354, 437135, 1219293, 1444714, 1605304, 1689402, 1717733 | 5 |
| Patient has a history of Obstructive Sleep Apnea | G47.33 | 2 |
| Patient has a history of HIV or is taking an antiviral medication | B20 | 1 |
| Patient has a history of Pneumonectomy | Z90.2 | 3.5 |
| Patient has a history of Hyponatremia | E87.1 | 4 |
| Patient has a history of Spinal Cord Injury | S14, S14.0, S14.0XXA, S14.0XXD, S14.0XXS, S14.1, S14.10, S14.101, S14.101A, S14.101D, S14.101S, S14.102, S14.102A, S14.102D, S14.102S, S14.103, S14.103A, S14.103D, S14.103S, S14.104, S14.104A, S14.104D, S14.104S, S14.105, S14.105A, S14.105D, S14.105S, S14.106, S14.106A, S14.106D, S14.106S, S14.107, S14.107A, S14.107D, S14.107S, S14.108, S14.108A, S14.108D, S14.108S, S14.109, S14.109A, S14.109D, S14.109S, S14.11, S14.111, S14.111A, S14.111D, S14.111S, S14.112, S14.112A, S14.112D, S14.112S, S14.113, S14.113A, S14.113D, S14.113S, S14.114, S14.114A, S14.114D, S14.114S, S14.115, S14.115A, S14.115D, S14.115S, S14.116, S14.116A, S14.116D, S14.116S, S14.117, S14.117A, S14.117D, S14.117S, S14.118, S14.118A, S14.118D, S14.118S, S14.119, S14.119A, S14.119D, S14.119S, S14.12, S14.121, S14.121A, S14.121D, S14.121S, S14.122, S14.122A, S14.122D, S14.122S, S14.123, S14.123A, S14.123D, S14.123S, S14.124, S14.124A, S14.124D, S14.124S, S14.125, S14.125A, S14.125D, S14.125S, S14.126, S14.126A, S14.126D, S14.126S, S14.127, S14.127A, S14.127D, S14.127S, S14.128, S14.128A, S14.128D, S14.128S, S14.129, S14.129A, S14.129D, S14.129S, S14.13, S14.131, S14.131A, S14.131D, S14.131S, S14.132, S14.132A, S14.132D, S14.132S, S14.133, S14.133A, S14.133D, S14.133S, S14.134, S14.134A, S14.134D, S14.134S, S14.135, S14.135A, S14.135D, S14.135S, S14.136, S14.136A, S14.136D, S14.136S, S14.137, S14.137A, S14.137D, S14.137S, S14.138, S14.138A, S14.138D, S14.138S, S14.139, S14.139A, S14.139D, S14.139S, S14.14, S14.141, S14.141A, S14.141D, S14.141S, S14.142, S14.142A, S14.142D, S14.142S, S14.143, S14.143A, S14.143D, S14.143S, S14.144, S14.144A, S14.144D, S14.144S, S14.145, S14.145A, S14.145D, S14.145S, S14.146, S14.146A, S14.146D, S14.146S, S14.147, S14.147A, S14.147D, S14.147S, S14.148, S14.148A, S14.148D, S14.148S, S14.149, S14.149A, S14.149D, S14.149S, S14.15, S14.151, S14.151A, S14.151D, S14.151S, S14.152, S14.152A, S14.152D, S14.152S, S14.153, S14.153A, S14.153D, S14.153S, S14.154, S14.154A, S14.154D, S14.154S, S14.155, S14.155A, S14.155D, S14.155S, S14.156, S14.156A, S14.156D, S14.156S, S14.157, S14.157A, S14.157D, S14.157S, S14.158, S14.158A, S14.158D, S14.158S, S14.159, S14.159A, S14.159D, S14.159S, S14.2, S14.2XXA, S14.2XXD, S14.2XXS, S14.3, S14.3XXA, S14.3XXD, S14.3XXS, S14.4, S14.4XXA, S14.4XXD, S14.4XXS, S14.5, S14.5XXA, S14.5XXD, S14.5XXS, S14.8, S14.8XXA, S14.8XXD, S14.8XXS, S14.9, S14.9XXA, S14.9XXD, S14.9XXS, S24, S24.0, S24.0XXA, S24.0XXD, S24.0XXS, S24.1, S24.10, S24.101, S24.101A, S24.101D, S24.101S, S24.102, S24.102A, S24.102D, S24.102S, S24.103, S24.103A, S24.103D, S24.103S, S24.104, S24.104A, S24.104D, S24.104S, S24.109, S24.109A, S24.109D, S24.109S, S24.11, S24.111, S24.111A, S24.111D, S24.111S, S24.112, S24.112A, S24.112D, S24.112S, S24.113, S24.113A, S24.113D, S24.113S, S24.114, S24.114A, S24.114D, S24.114S, S24.119, S24.119A, S24.119D, S24.119S, S24.13, S24.131, S24.131A, S24.131D, S24.131S, S24.132, S24.132A, S24.132D, S24.132S, S24.133, S24.133A, S24.133D, S24.133S, S24.134, S24.134A, S24.134D, S24.134S, S24.139, S24.139A, S24.139D, S24.139S, S24.14, S24.141, S24.141A, S24.141D, S24.141S, S24.142, S24.142A, S24.142D, S24.142S, S24.143, S24.143A, S24.143D, S24.143S, S24.144, S24.144A, S24.144D, S24.144S, S24.149, S24.149A, S24.149D, S24.149S, S24.15, S24.151, S24.151A, S24.151D, S24.151S, S24.152, S24.152A, S24.152D, S24.152S, S24.153, S24.153A, S24.153D, S24.153S, S24.154, S24.154A, S24.154D, S24.154S, S24.159, S24.159A, S24.159D, S24.159S, S24.2, S24.2XXA, S24.2XXD, S24.2XXS, S24.3, S24.3XXA, S24.3XXD, S24.3XXS, S24.4, S24.4XXA, S24.4XXD, S24.4XXS, S24.8, S24.8XXA, S24.8XXD, S24.8XXS, S24.9, S24.9XXA, S24.9XXD, S24.9XXS, S34, S34.0, S34.01, S34.01XA, S34.01XD, S34.01XS, S34.02, S34.02XA, S34.02XD, S34.02XS, S34.1, S34.10, S34.101, S34.101A, S34.101D, S34.101S, S34.102, S34.102A, S34.102D, S34.102S, S34.103, S34.103A, S34.103D, S34.103S, S34.104, S34.104A, S34.104D, S34.104S, S34.105, S34.105A, S34.105D, S34.105S, S34.109, S34.109A, S34.109D, S34.109S, S34.11, S34.111, S34.111A, S34.111D, S34.111S, S34.112, S34.112A, S34.112D, S34.112S, S34.113, S34.113A, S34.113D, S34.113S, S34.114, S34.114A, S34.114D, S34.114S, S34.115, S34.115A, S34.115D, S34.115S, S34.119, S34.119A, S34.119D, S34.119S, S34.12, S34.121, S34.121A, S34.121D, S34.121S, S34.122, S34.122A, S34.122D, S34.122S, S34.123, S34.123A, S34.123D, S34.123S, S34.124, S34.124A, S34.124D, S34.124S, S34.125, S34.125A, S34.125D, S34.125S, S34.129, S34.129A, S34.129D, S34.129S, S34.13, S34.131, S34.131A, S34.131D, S34.131S, S34.132, S34.132A, S34.132D, S34.132S, S34.139, S34.139A, S34.139D, S34.139S, S34.2, S34.21, S34.21XA, S34.21XD, S34.21XS, S34.22, S34.22XA, S34.22XD, S34.22XS, S34.3, S34.3XXA, S34.3XXD, S34.3XXS, S34.4, S34.4XXA, S34.4XXD, S34.4XXS, S34.5, S34.5XXA, S34.5XXD, S34.5XXS, S34.6, S34.6XXA, S34.6XXD, S34.6XXS, S34.8, S34.8XXA, S34.8XXD, S34.8XXS, S34.9, S34.9XXA, S34.9XXD, S34.9XXS | 4.5 |
| Patient has a history of Pacemaker | Z95, Z95.0, Z95.810, Z95.811, Z95.812, Z45.010 | 4.5 |
| Patient has a history of Malignant Hyperthermia | T88.3XXA, Z84.89 | 5 |
| Patient has a history of Stroke | G45, G45.0, G45.1, G45.2, G45.3, G45.4, G45.8, G45.9, G52.7, I63, I63.0, I63.00, I63.01, I63.011, I63.012, I63.013, I63.019, I63.02, I63.03, I63.031, I63.032, I63.033, I63.039, I63.09, I63.1, I63.10, I63.11, I63.111, I63.112, I63.113, I63.119, I63.12, I63.13, I63.131, I63.132, I63.133, I63.139, I63.19, I63.2, I63.20, I63.21, I63.211, I63.212, I63.213, I63.219, I63.22, I63.23, I63.231, I63.232, I63.233, I63.239, I63.29, I63.3, I63.30, I63.31, I63.311, I63.312, I63.313, I63.319, I63.32, I63.321, I63.322, I63.323, I63.329, I63.33, I63.331, I63.332, I63.333, I63.339, I63.34, I63.341, I63.342, I63.343, I63.349, I63.39, I63.4, I63.40, I63.41, I63.411, I63.412, I63.413, I63.419, I63.42, I63.421, I63.422, I63.423, I63.429, I63.43, I63.431, I63.432, I63.433, I63.439, I63.44, I63.441, I63.442, I63.443, I63.449, I63.49, I63.5, I63.50, I63.51, I63.511, I63.512, I63.513, I63.519, I63.52, I63.521, I63.522, I63.523, I63.529, I63.53, I63.531, I63.532, I63.533, I63.539, I63.54, I63.541, I63.542, I63.543, I63.549, I63.59, I63.6, I63.8, I63.81, I63.89, I63.9, Z86.73, Z86.79 | 5 |
| Patient has a history of Neuromuscular Disease | G35, G35.D, G35.B0, G35.A | 5 |
| Patient has a history of ICD Implant | Z95.810 | 5 |
| Patient has a history of Congenital Heart Disease | Q04.8, Q20, Q20.0, Q20.1, Q20.2, Q20.3, Q20.4, Q20.5, Q20.6, Q20.8, Q20.9, Q21, Q21.0, Q21.1, Q21.10, Q21.11, Q21.12, Q21.13, Q21.14, Q21.15, Q21.16, Q21.19, Q21.2, Q21.20, Q21.21, Q21.22, Q21.23, Q21.3, Q21.4, Q21.8, Q21.9, Q22, Q22.0, Q22.1, Q22.2, Q22.3, Q22.4, Q22.5, Q22.6, Q22.8, Q22.9, Q23, Q23.0, Q23.1, Q23.2, Q23.3, Q23.4, Q23.8, Q23.81, Q23.82, Q23.88, Q23.9, Q24, Q24.0, Q24.1, Q24.2, Q24.3, Q24.4, Q24.5, Q24.6, Q24.8, Q24.9, Q25, Q25.0, Q25.1, Q25.2, Q25.21, Q25.29, Q25.3, Q25.4, Q25.40, Q25.41, Q25.42, Q25.43, Q25.44, Q25.45, Q25.46, Q25.47, Q25.48, Q25.49, Q25.5, Q25.6, Q25.7, Q25.71, Q25.72, Q25.79, Q25.8, Q25.9, Q26, Q26.0, Q26.1, Q26.2, Q26.3, Q26.4, Q26.5, Q26.6, Q26.8, Q26.9, Q27, Q27.0, Q27.1, Q27.2, Q27.3, Q27.30, Q27.31, Q27.32, Q27.33, Q27.34, Q27.39, Q27.4, Q27.8, Q27.9, Q28, Q28.0, Q28.1, Q28.2, Q28.3, Q28.8, Q28.9 | 5 |
| Patient has a history of End Stage Renal Disease | N18.6 | 5 |
| Patient has a history of Dialysis | Z99.2 | 5 |
| Patient has a history of Heart Transplant | Z94.1 | 5 |
| Patient has a history of hydrocephalus | G91.2, G91, G91.0, G91.1, G91.9, G91.8 | 2 |
| Patient has a history of cognitive dysfunction | F09 | 2 |
| Patient has a history of vasovaga syncope | R55 | 1 |
| Patient has a history of swelling of feet or ankles | M25.471, M25.474, M25.475, M25.472, M25.473, M25.476 | 1 |
| Patient has a history of shortness of breath | R06.02, R06, R06.01, R06.03, R06.09, R06.00, R06.0, R06.89 | 2 |
| Patient has a history of sarcoidosis | D86.85, D86, D86.0 | 5 |
| Patient has a history of VT/SVT | I47.20, I47, I47.10, I47.1, I47.11, I47.19 | 2 |
| Patient has a history of mediastinitis | J98.59, J98.51 | 5 |
| Patient has a history of pleural effusion | J90, J91.8, J94.8 | 3 |
| Patient has a history of pericardial effusion | I31.39, I31.3, I31.2, I31.31 | 5 |
| Patient has a history of atrial fibrillation | I48.91, I48.20, I48.92, I48.19 | 3 |
| Patient has a history of chordoma | C41.2 | 1 |
| Patient has a history of AV Block | I44.2, I44, I44.0, I44.1, I44.3, I44.30, I44.39 | 3 |
| Patient has a history of Parkinson's Disease or is taking an antiparkinsonian medication | G20.A1, G20.A2, G20.B1, G20.B2, G90.3 | 1 |
| Patient has a history of vocal fold atrophy | J38.3, J38, J38.1, J38.0, J38.2, J38.00, J38.01, J38.02 | 2 |
| Patient has a history of cervical dystonia | G24.3, G24.8 | 2 |
| Patient has a history of kidney transplant | Z94.0 | 2 |
| Patient has a history of hypertrophic cardiomyopathy | I42.1 | 5 |
| Patient has a history of cystic fibrosis | E84.9, E84, E84.0, E84.1, E84.11, E84.19, E84.8 | 4 |
| Patient has a history of carotid stenosis | I65.23, I65.21, I65.22, I65.29 | 2 |
| Patient has a history of POTS | G90.A | 2 |
| Patient has a history of paraplegia | G82.20, G82.2, G82.21, G82.22, G82 | 5 |
| Patient has a history of pressure ulcer | L89.109, L89.159, L89.151, L89.152, L89.153, L89.154, L89.150, L89.100, L89.101, L89.102, L89.103, L89.104, L89.106, L89.110, L89.111, L89.112, L89.113, L89.114, L89.116, L89.119, L89.120, L89.121, L89.123, L89.122, L89.126, L89.129, L89.130, L89.131, L89.132, L89.133, L89.134, L89.136, L89.139, L89.140, L89.141, L89.142, L89.143, L89.144, L89.146, L89.149 | 2 |
| Patient has a history of impaired mobility | Z74.09, Z74, Z74.0, Z74.01, Z74.8, Z74.1, Z74.9 | 3 |
| Patient has a history of tricuspid atresia | Q20.4, Q22.4 | 5 |
| Patient has a history of cyanosis | R23.0 | 5 |
| Patient has a history of adult congenital heart disease | Q24.9 | 5 |
| Patient has a history of transposition of great arteries | Q20.3, Q20.4, Q20.5 | 5 |
| Patient has a history of sick sinus syndrome | I49.5 | 5 |
| Patient has a history of coarctation of aorta | Q25.1 | 5 |
| Patient has a history of left ventricular dysfunction | I51.89, I51.9 | 4 |
| Patient has a history of fontan circulation or procedure | Q20.8, Z87.74 | 5 |
| Patient has an active order for EKG or A1C | presence of active order | 10 |
| Patient has a history of Myasthenia Gravis | G70, G70.0, G70.00, G70.01 | 6 |
| Patient has a history of coronary artery disease or is taking an antianginal agent | I25.1, I25.10, I25.11, I25.111, I25.112, I25.118, I25.119, I25.2, I25.3, I25.4, I25.41, I25.42, I25.5, I25.6, I25.7, I25.70, I25.701, I25.700, I25.702, I25.708, I25.709, I25.71, I25.710, I25.711, I25.712, I25.718, I25.719, I25.72, I25.721, I25.722, I25.728, I25.729, I25.73, I25.731, I25.732, I25.738, I25.739, I25.75, I25.751, I25.752, I25.758, I25.759, I25.76, I25.761, I25.762, I25.768, I25.769, I25.79, I25.791, I25.792, I25.798, I25.799, I25.8, I25.81, I25.811, I25.812, I25.82, I25.83, I25.84, I25.85, I25.89, I25.9, I21, I21.0, I21.1, I21.2, I21.3, I21.4, I21.01, I21.02, I22, I22.0, I22.2, I22.8, I22.9 | 3 |
| Patient is taking an anticoagulant | institution-specific grouper for this medication class | 6 |
| Patient is taking a beta blocker | institution-specific grouper for this medication class | 0.5 |
| Patient is taking a calcium channel blocker | institution-specific grouper for this medication class | 0.5 |
| Patient is taking an antiarrhythmic | institution-specific grouper for this medication class | 0.5 |
| Patient is taking a corticosteroid | institution-specific grouper for this medication class | 0.5 |
| Patient is taking a diuretic | institution-specific grouper for this medication class | 2 |
| Patient is taking a cardiovascular medication | institution-specific grouper for this medication class | 4 |
| Patient is taking a antimyasthenic | institution-specific grouper for this medication class | 4 |
| Patient is taking a cardiotonic medication | institution-specific grouper for this medication class | 4 |
| Patient is taking a respiratory medication | institution-specific grouper for this medication class | 3 |
| Patient has a dependence on supplemental oxygen | Z99.81 | 2 |
| Patient has an insulin pump | Z96.41 | 5.5 |
